# The benefit of metformin in the treatment of pediatric non-alcoholic fatty liver disease: a systematic review and meta-analysis of randomized controlled trials

**DOI:** 10.1007/s00431-023-05169-9

**Published:** 2023-08-28

**Authors:** Nikolaos Gkiourtzis, Panagiota Michou, Maria Moutafi, Agni Glava, Konstantinos Cheirakis, Aristeidis Christakopoulos, Eleni Vouksinou, Maria Fotoulaki

**Affiliations:** 1 4th Department of Pediatrics, Papageorgiou General Hospital, School of Medicine, Faculty of Health Sciences, Aristotle University of Thessaloniki, Ring Road, Municipality of Pavlou Mela, Area N. Evkarpia, Thessaloniki, 56429 Greece; 2 Department of Pediatrics, Gennimatas General Hospital of Thessaloniki, Thessaloniki, Greece

**Keywords:** NAFLD, Fatty liver, Metformin, Children, Systematic review

## Abstract

This is the first meta-analysis of the available literature about the efficacy of metformin exclusively in pediatric patients with non-alcoholic fatty liver disease (NAFLD). We conducted a systematic literature search through major electronic databases till March 12, 2023, investigating the efficacy and safety of metformin in pediatric NAFLD. Weighted mean difference (WD) and standard deviation (SD) were used for continuous outcomes. In total, 4 randomized controlled trials (RCTs) with 309 pediatric patients with NAFLD were included in the meta-analysis. Metformin could not reach a statistically significant improvement in alanine aminotransferase (ALT) levels [(ALT: WMD =  − 1.55 IU/L, 95% CI: − 5.38 to 2.28, *I*^2^ = 16%, *p* = 0.43), but had a statistically significant impact (*p* < 0.05) in insulin and HOMA-IR regulation, triglycerides, and high-density lipoprotein level improvement.

*Conclusion*: According to the data of this meta-analysis, treatment with metformin failed to statistically improve liver enzymes but may be beneficial in the improvement of lipid parameters and insulin metabolism regulation in pediatric patients with NAFLD. As there are not enough available studies in the literature, the influence of metformin on liver ultrasonography or histology in pediatric NAFLD should be further analyzed in future studies.

**Table:** 

**What is Known:** *• Lifestyle modification with weight loss through physical activity and dietary modification is the recommended treatment option for pediatric NAFLD.* *• Metformin may reduce steatosis on ultrasound and may have a beneficial role in liver histology collated with insulin resistance improvement.*	
**What is New:** *• Metformin may improve insulin sensitivity and lipid parameters in children with obesity and NAFLD.* *• Metformin does not have a significant effect on transaminase levels in children with obesity and NAFLD.*

**Supplementary Information:**

The online version contains supplementary material available at 10.1007/s00431-023-05169-9.

## Introduction

Non-alcoholic fatty liver disease (NAFLD) is the most common pediatric chronic liver disease, with its prevalence increasing among overweight and obese children and its incidence rising over time [1, 2]. NAFLD included a wide spectrum of manifestations, from simple hepatic steatosis to advanced non-alcoholic steatohepatitis (NASH) with histologic features of inflammation and fibrosis, leading to end-stage liver disease [2, 3]. Initial screening for the diagnosis of NAFLD includes the exclusion of secondary causes of liver steatosis and the use of two times the sex-specific alanine aminotransferase (ALT) (≥ 52 U/L for boys and ≥ 44 U/L for girls) in children ≥ 10 years, with body mass index (BMI) ≥ 85th and < 94th (overweight) or ≥ 95th percentile (obese), with a sensitivity of 88%, but low specificity of 26% [1, 2]. The “gold standard” method for diagnosis and disease staging is liver biopsy [2–4].

The currently recommended management option for pediatric NAFLD is lifestyle changes with weight loss through physical activity and dietary modification [2, 4]. Recent studies examined the use of metformin in children with NAFLD and showed promising results, but still controversial [5–8]. Metformin has been shown to lead to a reduction of steatosis on ultrasound (US) having a possible beneficial role in liver histology collated with insulin resistance improvement [8]. According to experimental studies, the positive effect of metformin in NAFLD is related to the activation of adenosine monophosphate-activated protein kinase (AMPK) that regulates the metabolic patterns of glucose and lipids [9, 10]. Another hypothesis is that metformin oral administration is associated with changes in the gut microbiota and lower translocation of bacterial endotoxins resulting in insulin resistance improvement in patients with NAFLD [9, 11]. Finally, results of a recent meta-analysis in adult patients with NASH showed significant improvement in anthropometric parameters, insulin resistance, and lipid parameters in the group of metformin, but no improvement was revealed in liver histology parameters [12]. Taking into account the key characteristics of children, we conducted a systematic review and meta-analysis of randomized controlled trials (RCTs) to clarify the effectiveness of metformin in the management of pediatric NAFLD.

## Materials and methods

### Study registration and search methodology

This meta-analysis was conducted according to the Preferred Reporting Items for Systematic Reviews and Meta-analyses (PRISMA) guidelines and the Cochrane Handbook for Systematic Reviews of Interventions [13, 14]. A prespecified protocol has been registered in OSF (https://osf.io/jmbz8). Our search strategy was based on the publications in the main medical e-databases (PubMed/MEDLINE and Scopus) (Appendix Table 1) including relevant terms for NAFLD, children, and metformin. There were no limitations for publication year. Studies published until March 12, 2023 were included in our meta-analysis. We screened all the references from the included studies for additional studies. Clinicaltrials.gov, PROSPERO, OSF, conference papers, and grey literature were searched to identify relevant unpublished or published studies and trials to avoid duplication. Finally, only studies published in English language were included in our systematic review and meta-analysis.

### Eligibility criteria

In this systematic review, we included studies — observational or clinical trials — conducted on pediatric patients with NAFLD, while in the meta-analysis, we included only placebo-controlled RCTs. The research question (PICO) was defined using the following criteria [15]: articles published in English language with no limitation on the publication year; children and adolescents with NAFLD; the diagnosis of NAFLD was made by US, magnetic resonance imaging, computed tomography, or liver biopsy [2]; metformin with or without co-administration of other active interventions and placebo was administered orally to the subjects of the intervention and control groups for at least 8 weeks accordingly; the primary outcomes were mean change in alanine aminotransferase (ALT); the secondary outcomes were changes in aspartate aminotransferase (AST), body mass index (BMI) serum lipids [total cholesterol (TCHOL), high-density lipoprotein (HDL), low-density lipoprotein (LDL), triglycerides (TG)], fasting blood glucose (FBG), fasting blood insulin, homeostasis model assessment-insulin resistance (HOMA-IR), and US evaluation indicative of steatosis; studies that involved patients with chronic liver conditions or adults were excluded.

### Study procedure

Two authors (PM and MM) independently performed the search of the literature extracting and importing all records in a reference management tool (rayan.qcri.org), and duplicates were removed [16]. Then, they independently screened the title and abstract of all the retrieved records. The remaining studies were assessed independently by full-text reading, and in case of disagreements, a third reviewer (NG) made the final decision. Finally, two reviewers (PM and MM) independently extracted the data (publication year, study location, identification number, number of patients included in each study, treatment and patients’ characteristics, and duration of follow-up) of the eligible studies into a pre-specified data extraction form. If any study missed data, corresponding authors were contacted to obtain sufficient data.

### Quality assessment

The risk of bias was assessed independently by two examiners (NG and AG) using the revised Cochrane risk-of-bias (RoB 2.0) tool for randomized trials for each outcome [14, 17]. RoB tool consists of five domains: detection bias; attrition bias; reporting bias, and the overall assessment of RoB. Studies were graded as low risk when all domains were classified as “*low risk*”, “*some concerns*,” or “*high risk*” in studies which had one domain classified as “high risk,” or three domains were classified as *some concerns*.

### Outcome measurements

The primary outcome was the mean change in ALT levels after treatment with metformin. Secondary outcomes were mean changes in AST levels, lipid profile (TCHOL, LDL, HDL, TG), mean change in BMI, FBG, fasting blood insulin, HOMA-IR, and US improvement of steatosis after treatment with metformin. US improvement was assessed by US echogenicity grading score (grade 0: normal liver without steatosis; grade 1: mild steatosis; grade 2: moderate steatosis; grade 3: severe steatosis).

### Statistical analysis

Review manager software 5.4 (RevMan 5.4) was used for statistical analyses when data were available for at least two RCTs [14]. Mean values and standard deviations (SD) were used for quantitative data analysis. Mean changes in mean values between post-intervention and baseline values were measured and presented as weighted mean difference (WMD) for continuous outcomes. Qualitive data were analyzed using 95% confidence interval (95% CI). Heterogeneity between the studies was assessed using the *I*^2^ test. *I*^2^ value < 40% was set as low, 30–60% as moderate, 50–90% as substantial, and 75–100% as considerable [14]. When *I*^2^ was > 50%, the random effect model was applied. For the analyses, *p*-value < 0.05 was considered statistically significant. Finally, we conducted a leave-one-out analysis, omitting each study consecutively to explore its effect on the overall outcome [14].

## Results

### Search results

**Fig. 1:**
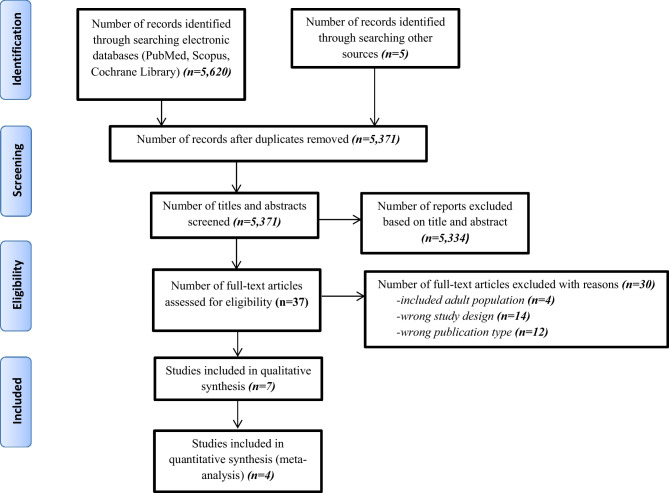
PRISMA 2009 flow diagram for study selection

In total, 5627 records were identified from our literature search. After duplicate removal and title and abstract screening, 37 studies remained for full-text assessment for eligibility. Finally, seven studies (five RCTs, a single-arm clinical trial and an observational study) were included in the systematic review (Fig. 1; Table 1) [6, 7, 18–22].

**Table 1:** Baseline characteristics of the pediatrics patients with NAFLD included in the systematic review and meta-analysis; non-alcoholic fatty liver disease

**Study ID**	**Identification number**	**Type of study**	**Country**	**Diagnosis**	**Intervention**	**Drug therapy (daily dose)**	**Follow-up period**	**Patients included/randomized**^a^	**Mean age (SD)**	**Male (%)**
Akcam 2011^b^	N/A	RCT	Turkey	Ultrasound	Metformin/diet/exercise	850 mg	6 months	22	12.0 (2.9)	50.0
					Diet/Exercise			22	11.3 (2.6)	45.5
Homaei 2022^b^	IRCT2017061334514N1	RCT	Iran	Ultrasound	Metformin/diet and exercise recommendation	1000 mg	3 months	50	11.8 (1.6)	34.2
					Placebo/diet and exercise recommendation			50	12.2 (1.5)	32.9
Lavine 2011^b^	NCT00063635	RCT	USA	Biopsy and histology	Metformin/vitamin E placebo/diet and exercise recommendation	1000 mg	28 months	57	13.4 (2.3)	82.5
					Vitamin E placebo/metformin placebo/diet and exercise recommendation			58	12.9 (2.6)	79.3
Nadeau 2009	M01RR00069	RCT	USA	Ultrasound	Metformin/diet/exercise	500 mg for 1 month, 1000 mg for 1 month and 1700 mg for 4 months	6 months	37	15.1 (10.8)	32.0
					Placebo/diet/exercise			13		38.0
Nobili 2008		Open-label observational pilot study	Italy	Biopsy and Histology	Metformin/diet/exercise	1500 mg	24 months	30	13.5 (5.5)	71.4
					Placebo/diet/exercise			30	13.4 (6.1)	68.9
Schwimmer 2005		Single-arm open-label pilot study	USA	Biopsy and histology	Metformin/diet recommendation	1000 mg	24 weeks	10	11.2 (2.7)	80.0
Shiashi Arani 2014^c^	IRCT21012421N1	RCT	Iran	Ultrasound	Metformin/diet and exercise recommendation (< 12 years of age)	1000 mg	2 months	36	8.24 (2.17)	41.7
					Metformin/diet and exercise recommendation (> 12 years of age)	1500 mg		28	13.53 (1.27)	64.3

*ID* identification, *RCT* randomized clinical trial, *SD* standard deviation

^a^Only for RCTs

^b^Only metformin and control groups included in this table

^c^Only metformin groups included in this table

### Baseline characteristics

The total randomized patients included in our meta-analysis were 309 from 4 RCTs (Table 1) [6, 19–21]. Participants’ mean age ranged from 8.24 ± 2.17 to 15.1 ± 10.8, and the male ratio (%) ranged from 32.9 to 82.5. The duration of RCT follow-up ranged between 3 and 28 months. The RCTs had an optional or mandatory co-intervention with lifestyle changes (nutritional counseling, diet, and exercise). Most studies used 1000 mg of metformin per day [18–20, 22], two studies used 1500 mg daily [7, 18], one study used 850 mg [6], and one study used 1700 mg of metformin [21]. Finally, most studies included only children with obesity and NAFLD [6, 18, 20–22], and only two studies included both overweight and obese patients [7, 19].

### Risk of bias in the included RCTs

**Fig. 2:**
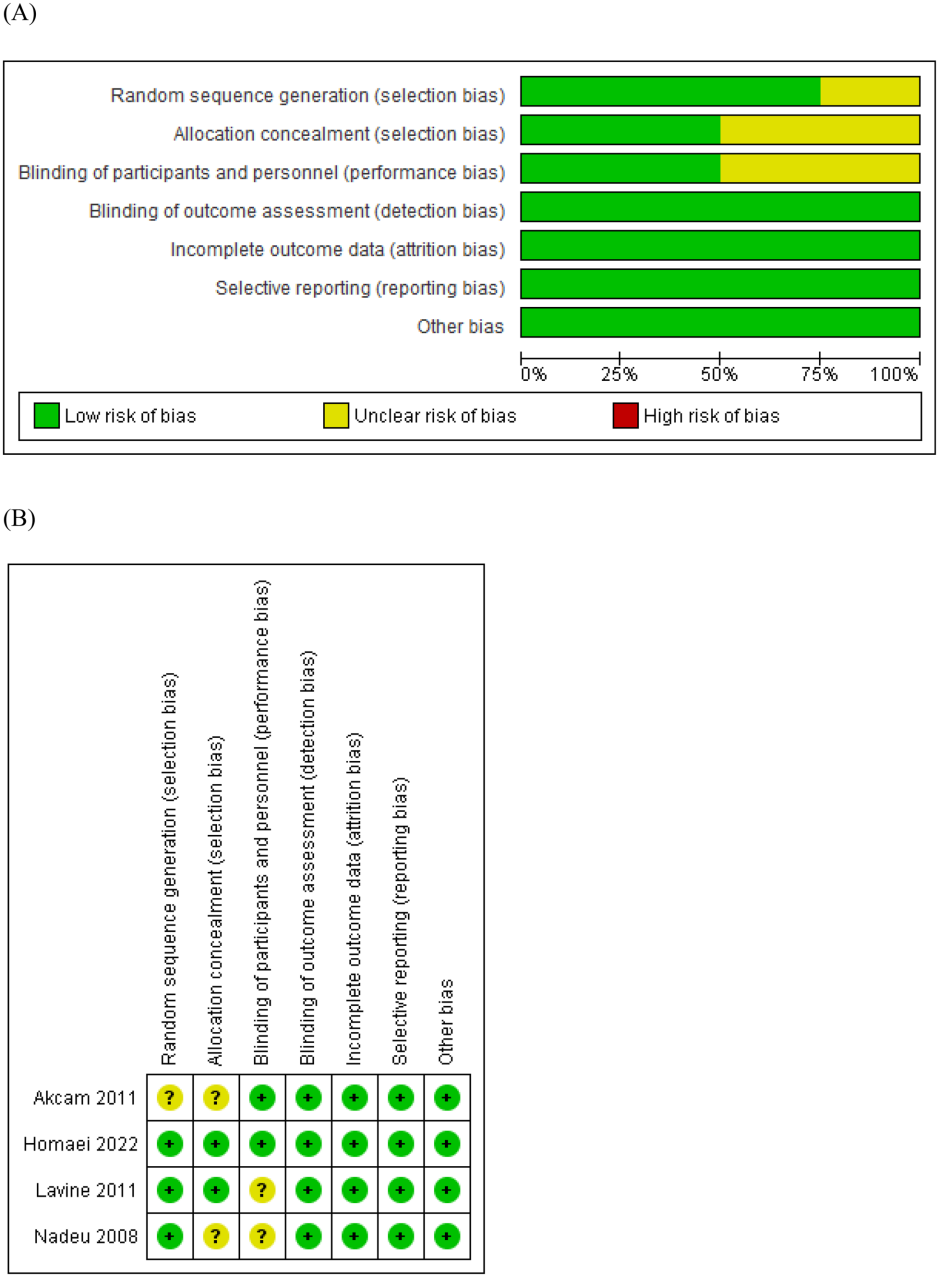
The assessment of the quality of clinical trials using Review Manager 5.4.1 (Revman 5.4.1). **A** Summary for the assessment of risk of bias. **B** Risk of bias; (+), low risk of bias; (?), unclear risk of bias; (−), high risk of bias

Three studies were evaluated as having “*some concerns*” about the risk of bias [6, 19, 21]. Only one study was evaluated as having a “*low*” risk of bias [20]. A summary of the risk of bias assessment is described in Fig. 2.

### Analysis of primary outcome

**Fig. 3:**
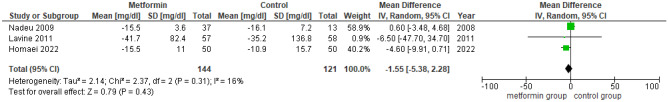
Impact of metformin on ALT, alanine aminotransferase

The effect of metformin on serum ALT levels was evaluated in three studies [19–21]. Metformin did not reveal a significant impact in ALT levels (WMD =  − 1.55 IU/L, 95% CI: − 5.38 to 2.28, *I*^2^ = 16%, *p* = 0.43) (Fig. 3).

### Analysis of secondary outcomes

**Fig. 4:**
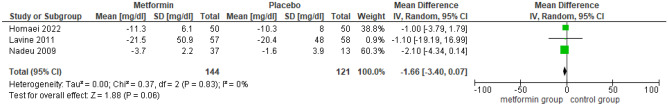
Impact of metformin on AST, aspartate aminotransferase

**Fig. 5:**
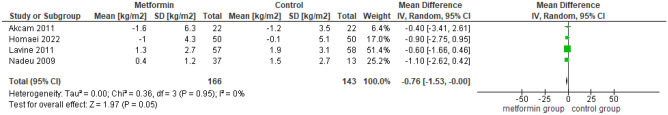
Impact of metformin on BMI, body mass index

**Fig. 6:**
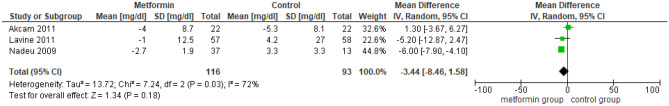
Impact of metformin on FBG, fasting blood glucose

**Fig. 7:**
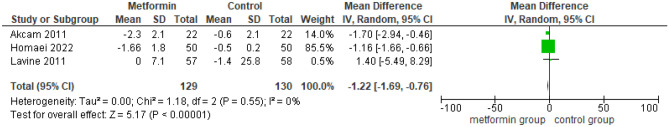
Impact of metformin on HOMA-IR, homeostasis model assessment-insulin resistance

**Fig. 8:**
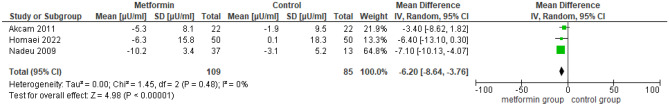
Impact of metformin on insulin

**Fig. 9:**
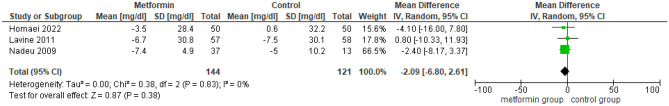
Impact of metformin on TCHOL, total cholesterol

**Fig. 10:**
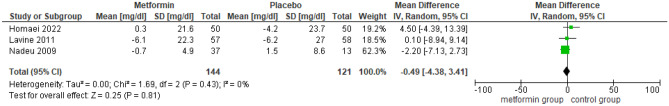
Impact of metformin on LDL, low-density lipoprotein

**Fig. 11:**
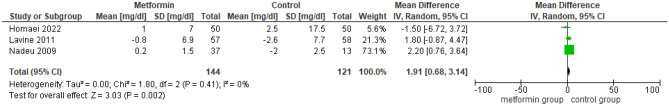
Impact of metformin on HDL, high-density lipoprotein

**Fig. 12:**
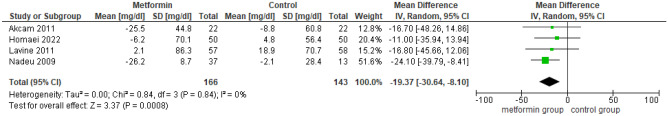
Impact of metformin on TG, triglycerides

**Fig. 13:**
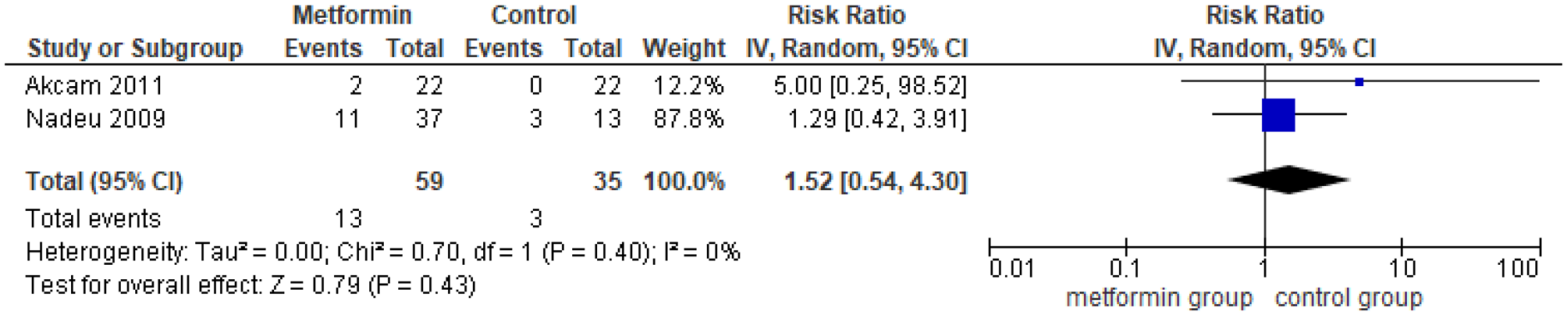
Impact of metformin on the risk for gastrointestinal side effects

Treatment with metformin showed no improvement in AST levels, BMI, and FBG levels (Figs. 4, 5, and 6). Metformin revealed a significant insulin regulation impact with statistically significant improvement in HOMA-IR (WMD =  − 1.22, 95% CI: − 1.69 to − 0.76, *I*^2^ = 0%, *p* < 0.00001) and insulin levels (WMD =  − 6.20, 95% CI: − 8.64 to − 3.76, *I*^2^ = 0%, *p* < 0.00001) (Figs. 7 and 8). Regarding serum lipid concentration, no significant change was detected in TCHOL and LDL levels (Figs. 9 and 10). Conversely, metformin revealed a significant difference in HDL (WMD =  − 1.91, 95% CI: 0.68 to 3.14, *I*^2^ = 0%, *p* = 0.002) and TG levels (WMD = 19.37, 95% CI: − 30.64 to − 8.10, *I*^2^ = 0%, *p* = 0.0008) (Figs. 11 and 12). US and histopathological data from three studies could not be synthesized and analyzed due to the different data demonstration [19–21]. Finally, only two studies evaluated the impact of metformin on the risk of gastrointestinal disorders (Fig. 13) [6, 21]. No increased risk of gastrointestinal disorders was revealed after treatment with metformin compared to the control group.

### Leave-one-out analysis

We carried out a leave-one-out sensitivity analysis for each outcome excluding each study separately to investigate any significant changes in the estimated effect with no significant changes noted for the primary outcome. Only after the exclusion of *Akcam 2011* a statistically significant improvement of FBG levels (*p* < 0.00001) and heterogeneity improvement (*I*^2^ = 0%) were revealed [6].

### Publication bias assessment

We could not assess publication bias with safety due to the small study number included in our meta-analysis (only four RCTs). For publication bias assessment, at least 10 studies in a meta-analysis are needed [23, 24]. Therefore, with a visual interpretation of the funnel plots (Appendix Figs. 2–12), the trim-and-fill method to estimate the effect size, and Egger’s test, we concluded that no increased risk for publication bias is raised regarding the studies included in this meta-analysis [23–25].

## Discussion

In this systematic review and meta-analysis, we evaluated the efficacy of metformin administration in pediatric patients with NAFLD. We included seven studies [6, 7, 18–22] with five RCTs [6, 18–21]. Treatment with metformin failed to significantly decrease transaminase levels but led to a statistically significant improvement of HOMA-IR, insulin, HDL, and TG levels after meta-analysis of included RCTs [6, 18–21].

NAFLD is the most common chronic liver disease, characterized by abnormal triglyceride accumulation in the liver cells after the exclusion of other causes of liver steatosis [2, 26, 27]. According to recent data, the incidence of NAFLD is estimated at 25% globally (8% in adolescents) [28, 29]. NAFLD spectrum is wide including NAFLD, NASH with/without cirrhosis, and hepatocellular carcinoma [2, 4]. International expert groups have reached a consensus to change the definition of NAFLD to metabolic-related fatty liver disease (MAFLD) due to pathogenesis and clinical presentation of this condition [30]. As no other medicine or supplement is proposed, lifestyle changes with diet and exercise are the only acceptable treatment option for pediatric NAFLD [2, 4].

The main benefits of metformin are the inhibition of hepatic gluconeogenesis and lipogenesis, improvement of lipid metabolism, fatty acid oxidation, and peripheral tissue insulin sensitivity [26–28, 31]. Metformin reduces the production of liver glucose and increases peripheral glucose uptake. It is an indirect activator of AMPK, an enzyme that according to experimental data leads to decreased intracellular fat accumulation and lipogenesis and inhibits lipid biosynthesis [9, 10].

The effect of metformin in patients with NAFLD has been previously explored [5, 8, 12, 26, 32–34]. Mann et al. conducted an extensive systematic review on lifestyle, dietary, and pharmacologic treatment options in pediatric NAFLD, but the lack of a meta-analysis led to a more arbitrary conclusion regarding the real benefit of metformin [8]. Theodoridis et al. conducted a network meta-analysis to compare a great variety of treatment options in pediatric NAFLD but did not include all the available studies evaluating intervention with metformin [5]. The rest of the systematic reviews and meta-analyses missed most of the data regarding pediatric NAFLD or focused mainly on adult patients with NAFLD [12, 26, 32–34].

To our knowledge, this is the first available systematic review and meta-analysis that provides the most updated data from recent studies, examining the effect of metformin exclusively in pediatric patients with NAFLD. We included four well-designed, placebo-controlled RCTs in this meta-analysis evaluating a great variety of clinical important endpoints [6, 19–21]. We followed an extensive literature search process through major medical databases, conference papers, and grey literature, screening all the available studies [14, 23]. As control groups in this meta-analysis, we used pediatric patients with NAFLD who received placebo for a pure comparison with the metformin group in order to draw safe results. RoB 2.0 tool was used for quality analysis, and Cochrane Handbook instructions were followed for the whole review process [14]. Moreover, low heterogeneity for all outcomes assessed was between the main advantages of this meta-analysis. Only one study had a duration of 3 months of treatment [20] with the remaining studies included in this meta-analysis ranging from 6 to 28 months of intervention [6, 19, 21]. Furthermore, no significant adverse events were reported in included studies, even if some patients dropped out. Finally, improvement of insulin and lipid parameters shows that metformin may have a significant role in the regulation of “parallel multiple-hit theory” pathways, targeting insulin resistance and excess lipid accumulation [35, 36].

The studies included in our systematic review and meta-analysis showed that metformin improves insulin sensitivity and may improve a variety of fatty liver parameters in children with NAFLD. Homaei et al. reported that the US fatty liver grade decreased more in the metformin group than in the other study groups (*p* < 0.05) [20]. In Nadeau et al., subjects with initial fatty liver in metformin group had significantly lower US fatty liver score than those in the placebo group [21]. In Shiashi Arani et al.’s trial, both metformin groups (1000 mg and 1500 mg per day groups) improved the US grade of fatty liver disease after 2 months of treatment (*p* < 0.05), but the group that received 1000 mg of metformin per day was the only one that showed statistically significant improvement after 4 months of treatment [18]. Lavine et al. showed significant improvement in hepatocellular ballooning scores after metformin administration (*p* = 0.04) in comparison to the control group, with no further significant improvement noted in other liver histology parameters [19]. In the observational study of Nobili et al., metformin did not appear more effective in steatosis and liver histology improvement than the control group [7]. Finally, Schwimmer et al. demonstrated significant improvements in liver fat in children with NAFLD measured by magnetic resonance spectroscopy (*p* < 0.01) [22].

Our systematic review and meta-analysis had some limitations that must be acknowledged. Despite the extensive literature search, only seven studies (four RCTs included in this meta-analysis) were eligible to be included in our study. Therefore, the small number of studies and included patients did not allow for any further subgroup analysis. The main limitation of our study is the lack of a meta-analysis regarding changes in liver histology after treatment with metformin, which is the gold standard for NAFLD diagnosis and monitoring [3, 37]. This is a common disadvantage of most studies on pediatric patients with NAFLD. Another reason is that we were unable to synthesize US results as they were presented with different scoring systems, either by the incidence of fatty liver [18, 21] or by liver grade after the intervention [20]. Another possible limitation of our study was that all included studies recommended lifestyle changes. Lifestyle change including diet and physical activity modifications during treatment with metformin is considered the first-line option in the management of pediatric NAFLD [2, 4]. Studies included in this meta-analysis did not use the same dosages of metformin per day or the same follow-up periods influencing the results. Regarding the risk of bias, most studies included in our meta-analysis were evaluated as having *some concerns* [6, 19, 21]. Finally, no statistically significant improvement was detected regarding transaminases, a surrogate marker for NAFLD activity and severity monitoring [2, 3].

According to the findings of our systematic review meta-analysis, apart from lifestyle changes, metformin, a classic insulin sensitizer seems to lead to some benefits in children with NAFLD, especially those with obesity, by improving some metabolic syndrome individual parameters. However, to confirm these findings, more studies are needed to examine the effect of metformin on US parameters and liver histology through serial liver biopsies.

In conclusion, metformin seems to be effective in improving insulin parameters as long as some lipid parameters in children with obesity and NAFLD. No significant effect on transaminase levels was noted. Analysis of liver US data from the studies included in the meta-analysis was not possible to draw safe conclusions. Future RCTs, examining the role of metformin on liver US and histology parameters should be conducted to confirm its beneficial effect on children with NAFLD.

## Supplementary Information

Below is the link to the electronic supplementary material.

Appendix Table 1. Literature search in the major medical databases (PubMed/MEDLINE and Scopus) (DOCX 14 KB)

Appendix Fig. 2. Funnel plot of the impact of metformin on ALT; alanine aminotransferase (DOCX 15 KB)

Appendix Fig. 3. Funnel plot of the impact of metformin on AST; aspartate aminotransferase (DOCX 15 KB)

Appendix Fig. 4. Funnel plot of the impact of metformin on BMI; body mass index (DOCX 15 KB)

Appendix Fig. 5. Funnel plot of the impact of metformin on FBG; fasting blood glucose (DOCX 15 KB)

Appendix Fig. 6. Funnel plot of the impact of metformin on HOMA-IR; homeostasis model assessment-insulin resistance (DOCX 15 KB)

Appendix Fig. 7. Funnel plot of the impact of metformin on insulin (DOCX 15 KB)

Appendix Fig. 8. Funnel plot of the impact of metformin on TCHOL; total cholesterol (DOCX 15 KB)

Appendix Fig. 9. Funnel plot of the impact of metformin on LDL; low-density lipoprotein (DOCX 15 KB)

Appendix Fig. 10. Funnel plot of the impact of metformin on HDL; high-density lipoprotein (DOCX 15 KB)

Appendix Fig. 11. Funnel plot of the impact of metformin on TG; triglycerides (DOCX 16 KB)

Appendix Fig. 12. Funnel plot of the impact of metformin on the risk for gastrointestinal side effects (DOCX 15 KB)

## Abbreviations

ALT: Alanine aminotransferase
AST: Aspartate aminotransferase
AMPK: Adenosine monophosphate-activated protein kinase
BMI: Body mass index
FBG: Fasting blood glucose
HDL: High-density lipoprotein
HOMA-IR: Homeostasis model assessment-insulin resistance
LDL: Low-density lipoprotein
MAFLD: Metabolic-related fatty liver disease
NAFLD: Non-alcoholic fatty liver disease
NASH: Non-alcoholic steatohepatitis
PICO: Population, Intervention, Comparison, and Outcome
PRISMA: Preferred Reporting Items for Systematic Reviews and Meta-analyses
RoB: Risk of bias
RCT: Randomized controlled trial
SD: Standard deviation
TCHOL: Total cholesterol
TG: Triglycerides
US: Ultrasound
WMD: Weighted mean difference

## References

[1] Sahota AK, Shapiro WL, Newton KP et al (2020) Incidence of nonalcoholic fatty liver disease in children: 2009–2018. Pediatrics 146:e20200771. 10.1542/peds.2020-0771 10.1542/peds.2020-0771 PMC7706110 33214329

[2] Vos MB, Abrams SH, Barlow SE et al (2017) NASPGHAN Clinical Practice Guideline for the Diagnosis and Treatment of Nonalcoholic Fatty Liver Disease in Children: Recommendations from the Expert Committee on NAFLD (ECON) and the North American Society of Pediatric Gastroenterology, Hepatology and N. J Pediatr Gastroenterol Nutr 64:319–334. 10.1097/MPG.0000000000001482 28107283 10.1097/MPG.0000000000001482 PMC5413933

[3] Vajro P, Lenta S, Socha P et al (2012) Diagnosis of nonalcoholic fatty liver disease in children and adolescents: position paper of the ESPGHAN Hepatology Committee. J Pediatr Gastroenterol Nutr 54:700–713. 10.1097/MPG.0b013e318252a13f 22395188 10.1097/MPG.0b013e318252a13f

[4] Chalasani N, Younossi Z, Lavine JE et al (2018) The diagnosis and management of nonalcoholic fatty liver disease: practice guidance from the American Association for the Study of Liver Diseases. Hepatology 67:328–357. 10.1002/hep.29367 28714183 10.1002/hep.29367

[5] Theodoridis X, Kalopitas G, Vadarlis A et al (2022) Comparative efficacy of different treatment modalities in the management of pediatric non-alcoholic fatty liver disease: a systematic review and network meta-analysis. Pharmacol Ther 240. 10.1016/j.pharmthera.2022.108294 10.1016/j.pharmthera.2022.108294 36183848

[6] Akcam M, Boyaci A, Pirgon O et al (2011) Therapeutic effect of metformin and vitamin E versus prescriptive diet in obese adolescents with fatty liver. Int J Vitam Nutr Res 81:398–406. 10.1024/0300-9831/a000086 22673924 10.1024/0300-9831/a000086

[7] Nobili V, Manco M, Ciampalini P et al (2008) Metformin use in children with nonalcoholic fatty liver disease: an open-label, 24-month, observational pilot study. Clin Ther 30:1168–1176. 10.1016/j.clinthera.2008.06.012 18640473 10.1016/j.clinthera.2008.06.012

[8] Mann JP, Tang GY, Nobili V, Armstrong MJ (2019) Evaluations of lifestyle, dietary, and pharmacologic treatments for pediatric nonalcoholic fatty liver disease: a systematic review. Clin Gastroenterol Hepatol 17:1457-1476.e7. 10.1016/J.CGH.2018.05.023 29857146 10.1016/j.cgh.2018.05.023

[9] Mazza A, Fruci B, Garinis GA et al (2012) The role of metformin in the management of NAFLD. Exp Diabetes Res 2012. 10.1155/2012/716404 10.1155/2012/716404 PMC3238361 22194737

[10] Anggreini P, Kuncoro H, Sumiwi SA, Levita J (2023) Role of the AMPK/SIRT1 pathway in non-alcoholic fatty liver disease (Review). Mol Med Rep 27. 10.3892/MMR.2022.12922 10.3892/mmr.2022.12922 PMC9827347 36562343

[11] Brandt A, Hernández-Arriaga A, Kehm R et al (2019) Metformin attenuates the onset of non-alcoholic fatty liver disease and affects intestinal microbiota and barrier in small intestine. Sci Rep 9:1 9:1–14. 10.1038/s41598-019-43228-0 10.1038/s41598-019-43228-0 PMC6491483 31040374

[12] Said A, Akhter A (2017) Meta-analysis of randomized controlled trials of pharmacologic agents in non-alcoholic steatohepatitis. Ann Hepatol 16:538–547. 10.5604/01.3001.0010.0284 28611274 10.5604/01.3001.0010.0284

[13] Moher D, Liberati A, Tetzlaff J, Altman DG (2009) Preferred reporting items for systematic reviews and meta-analyses: the PRISMA statement. Ann Intern Med 151(264–9):W64. 10.7326/0003-4819-151-4-200908180-00135 19622511 10.7326/0003-4819-151-4-200908180-00135

[14] Cumpston M, Li T, Page MJ et al (2019) Updated guidance for trusted systematic reviews: a new edition of the Cochrane Handbook for Systematic Reviews of Interventions. Cochrane Database Syst Rev 10:ED000142 10.1002/14651858.ED000142 PMC10284251 31643080

[15] Schardt C, Adams MB, Owens T et al (2007) Utilization of the PICO framework to improve searching PubMed for clinical questions. BMC Med Inform Decis Mak 7:16. 10.1186/1472-6947-7-16 17573961 10.1186/1472-6947-7-16 PMC1904193

[16] Ouzzani M, Hammady H, Fedorowicz Z, Elmagarmid A (2016) Rayyan-a web and mobile app for systematic reviews. Syst Rev 5:210. 10.1186/s13643-016-0384-4 27919275 10.1186/s13643-016-0384-4 PMC5139140

[17] Sterne JAC, Savović J, Page MJ et al (2019) RoB 2: a revised tool for assessing risk of bias in randomised trials. BMJ 366:l4898. 10.1136/bmj.l4898 10.1136/bmj.l4898 31462531

[18] Shiasi Arani K, Taghavi Ardakani A, Moazami Goudarzi R et al (2014) Effect of vitamin E and metformin on fatty liver disease in obese children- randomized clinical trial. Iran J Public Health 43:1417–1423 26060704 PMC4441895

[19] Lavine JE, Schwimmer JB, Van Natta ML et al (2011) Effect of vitamin E or metformin for treatment of nonalcoholic fatty liver disease in children and adolescents: the TONIC randomized controlled trial. JAMA 305:1659–1668. 10.1001/jama.2011.520 21521847 10.1001/jama.2011.520 PMC3110082

[20] Homaei A, Alhadad M, Arad B, Saffari F (2022) Effect of metformin or vitamin e on ultrasonographic grade and biochemical findings of children and adolescents with nonalcoholic fatty liver disease: a randomized clinical trial. J Compr Pediatr 13. 10.5812/compreped-123944

[21] Nadeau KJ, Ehlers LB, Zeitler PS, Love-Osborne K (2009) Treatment of non-alcoholic fatty liver disease with metformin versus lifestyle intervention in insulin-resistant adolescents. Pediatr Diabetes 10:5–13. 10.1111/j.1399-5448.2008.00450.x 18721166 10.1111/j.1399-5448.2008.00450.x

[22] Schwimmer JB, Middleton MS, Deutsch R, Lavine JE (2005) A phase 2 clinical trial of metformin as a treatment for non-diabetic paediatric non-alcoholic steatohepatitis. Aliment Pharmacol Ther 21:871–879. 10.1111/j.1365-2036.2005.02420.x 15801922 10.1111/j.1365-2036.2005.02420.x

[23] Lin L, Chu H, Murad MH et al (2018) Empirical comparison of publication bias tests in meta-analysis. J Gen Intern Med 33:1260–1267. 10.1007/s11606-018-4425-7 29663281 10.1007/s11606-018-4425-7 PMC6082203

[24] Dalton JE, Bolen SD, Mascha EJ (2016) Publication bias: the elephant in the review. Anesth Analg 123:812–813. 10.1213/ANE.0000000000001596 27636569 10.1213/ANE.0000000000001596 PMC5482177

[25] van Aert RCM, Wicherts JM, van Assen MALM (2019) Publication bias examined in meta-analyses from psychology and medicine: a meta-meta-analysis. PLoS ONE 14:e0215052 30978228 10.1371/journal.pone.0215052 PMC6461282

[26] Huang Y, Wang X, Yan C et al (2022) Effect of metformin on nonalcoholic fatty liver based on meta-analysis and network pharmacology. Medicine (United States) 101:E31437. 10.1097/MD.0000000000031437 10.1097/MD.0000000000031437 PMC9622616 36316840

[27] Lamoia TE, Shulman GI (2021) Cellular and molecular mechanisms of metformin action. Endocr Rev 42:77–96. 10.1210/ENDREV/BNAA023 32897388 10.1210/endrev/bnaa023 PMC7846086

[28] Clayton-Chubb D, Kemp W, Majeed A et al (2023) Understanding NAFLD: from case identification to interventions, outcomes, and future perspectives. Nutrients 15. 10.3390/NU15030687 10.3390/nu15030687 PMC9921401 36771394

[29] Anderson EL, Howe LD, Jones HE et al (2015) The prevalence of non-alcoholic fatty liver disease in children and adolescents: a systematic review and meta-analysis. PLoS One 10:e0140908. 10.1371/journal.pone.0140908 10.1371/journal.pone.0140908 PMC4626023 26512983

[30] Eslam M, Newsome PN, Sarin SK et al (2020) A new definition for metabolic dysfunction-associated fatty liver disease: an international expert consensus statement. J Hepatol 73:202–209. 10.1016/J.JHEP.2020.03.039 32278004 10.1016/j.jhep.2020.03.039

[31] J. Barbero-Becerra V, J. Santiago-Hernandez J, A. Villegas-Lopez F et al (2012) Mechanisms involved in the protective effects of metformin against nonalcoholic fatty liver disease. Curr Med Chem 19:2918–2923. 10.2174/092986712800672094 22519397 10.2174/092986712800672094

[32] Li Y, Liu L, Wang B et al (2013) Metformin in non-alcoholic fatty liver disease: a systematic review and meta-analysis. Biomed Rep 1:57. 10.3892/BR.2012.18 24648894 10.3892/br.2012.18 PMC3956897

[33] Jalali M, Rahimlou M, Mahmoodi M et al (2020) The effects of metformin administration on liver enzymes and body composition in non-diabetic patients with non-alcoholic fatty liver disease and/or non-alcoholic steatohepatitis: an up-to date systematic review and meta-analysis of randomized controlled trials. Pharmacol Res 159. 10.1016/j.phrs.2020.104799 10.1016/j.phrs.2020.104799 32278041

[34] Lian J, Fu J (2021) Efficacy of various hypoglycemic agents in the treatment of patients with nonalcoholic liver disease with or without diabetes: a network meta-analysis. Front Endocrinol (Lausanne) 12. 10.3389/FENDO.2021.649018/FULL 10.3389/fendo.2021.649018 PMC8024567 33841337

[35] Tilg H, Moschen AR (2010) Evolution of inflammation in nonalcoholic fatty liver disease: the multiple parallel hits hypothesis. Hepatology 52:1836–1846. 10.1002/HEP.24001 21038418 10.1002/hep.24001

[36] Bessone F, Razori MV, Roma MG (2019) Molecular pathways of nonalcoholic fatty liver disease development and progression. Cell Mol Life Sci 76:99–128. 10.1007/S00018-018-2947-0 30343320 10.1007/s00018-018-2947-0 PMC11105781

[37] Ma X, Liu S, Zhang J et al (2020) Proportion of NAFLD patients with normal ALT value in overall NAFLD patients: a systematic review and meta-analysis. BMC Gastroenterol 20. 10.1186/S12876-020-1165-Z 10.1186/s12876-020-1165-z PMC6961232 31937252

