# Supplementary material for: The benefit of metformin in the treatment of pediatric non-alcoholic fatty liver disease: a systematic review and meta-analysis of randomized controlled trials
Source: Eur J Pediatr. 2023 Aug 28;182(11):4795–806. doi: 10.1007/s00431-023-05169-9 (PMC10640492; doi:10.1007/s00431-023-05169-9)
Supplement: Supplementary file 1 — Appendix Table 1. Literature search in the major medical databases (PubMed/MEDLINE and Scopus) (DOCX 14 KB) [file 431_2023_5169_MOESM1_ESM.docx]

| **Suppl. Table 1. Literature search in major medical databases** |
| --- |
| **PubMed/MEDLINE (12/03/2023)** |
| (((non-alcoholic fatty liver disease) OR (steatosis)) AND (children)) AND (metformin) |
| **Results**: 83 |
| **Scopus (12/03/2023)** |
| ("non-alcoholic fatty liver disease") OR ("steatosis") AND ("children") AND ("metformin") |
| **Results**: 5,537 |
