# Supplementary figures and images for: The benefit of metformin in the treatment of pediatric non-alcoholic fatty liver disease: a systematic review and meta-analysis of randomized controlled trials
Source: Eur J Pediatr. 2023 Aug 28;182(11):4795–806. doi: 10.1007/s00431-023-05169-9 (PMC10640492; doi:10.1007/s00431-023-05169-9)

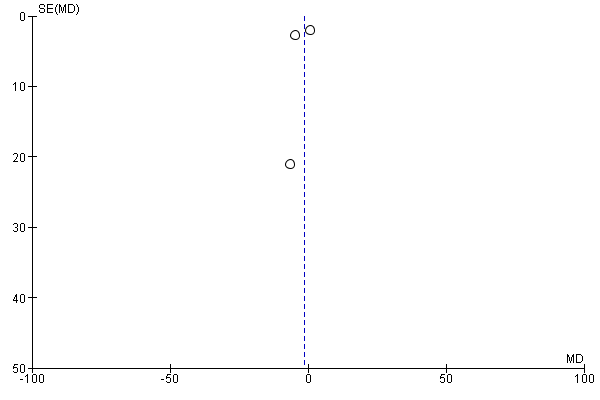

Supplement: Supplementary file 2 — Appendix Fig. 2. Funnel plot of the impact of metformin on ALT; alanine aminotransferase (DOCX 15 KB) [file 431_2023_5169_MOESM2_ESM.docx]

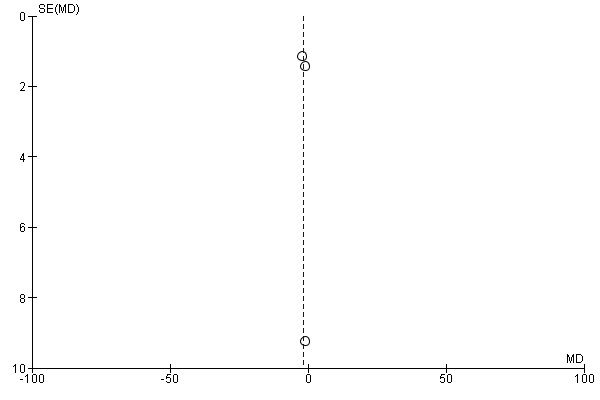

Supplement: Supplementary file 3 — Appendix Fig. 3. Funnel plot of the impact of metformin on AST; aspartate aminotransferase (DOCX 15 KB) [file 431_2023_5169_MOESM3_ESM.docx]

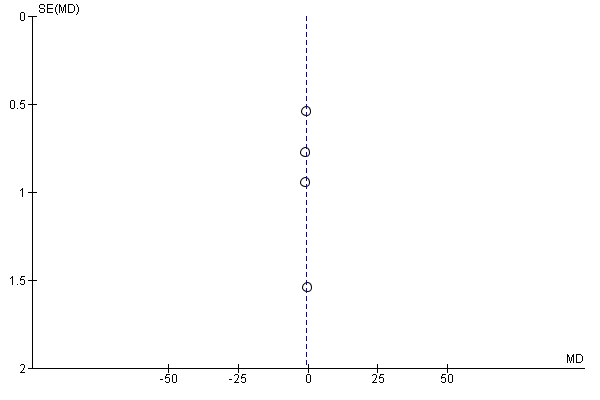

Supplement: Supplementary file 4 — Appendix Fig. 4. Funnel plot of the impact of metformin on BMI; body mass index (DOCX 15 KB) [file 431_2023_5169_MOESM4_ESM.docx]

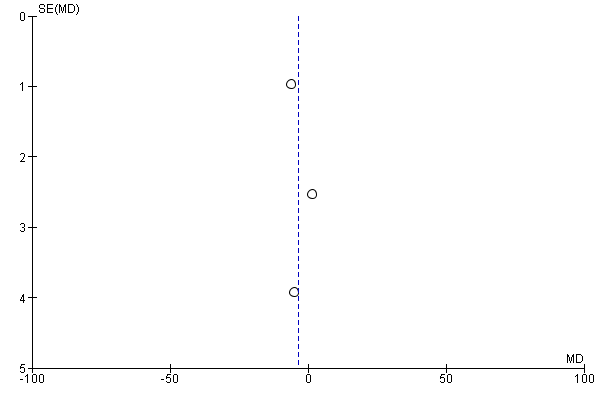

Supplement: Supplementary file 5 — Appendix Fig. 5. Funnel plot of the impact of metformin on FBG; fasting blood glucose (DOCX 15 KB) [file 431_2023_5169_MOESM5_ESM.docx]

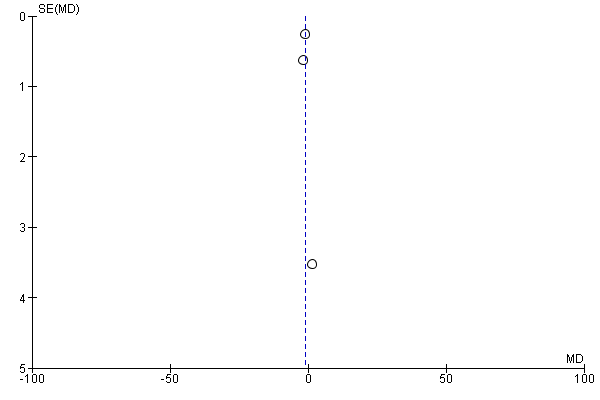

Supplement: Supplementary file 6 — Appendix Fig. 6. Funnel plot of the impact of metformin on HOMA-IR; homeostasis model assessment-insulin resistance (DOCX 15 KB) [file 431_2023_5169_MOESM6_ESM.docx]

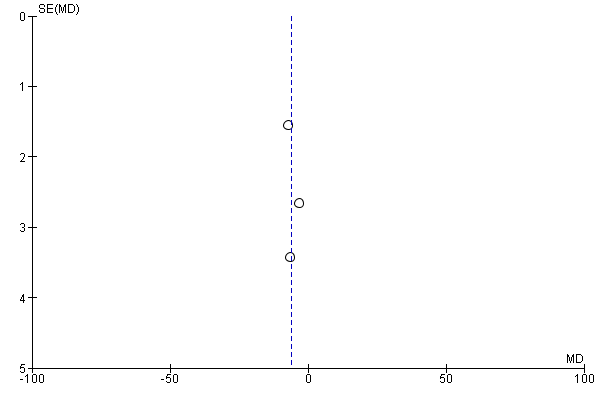

Supplement: Supplementary file 7 — Appendix Fig. 7. Funnel plot of the impact of metformin on insulin (DOCX 15 KB) [file 431_2023_5169_MOESM7_ESM.docx]

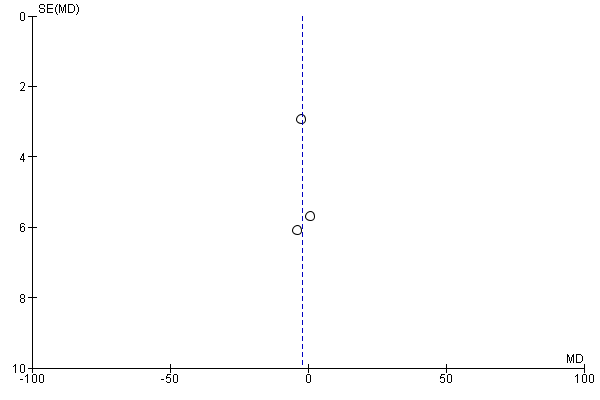

Supplement: Supplementary file 8 — Appendix Fig. 8. Funnel plot of the impact of metformin on TCHOL; total cholesterol (DOCX 15 KB) [file 431_2023_5169_MOESM8_ESM.docx]

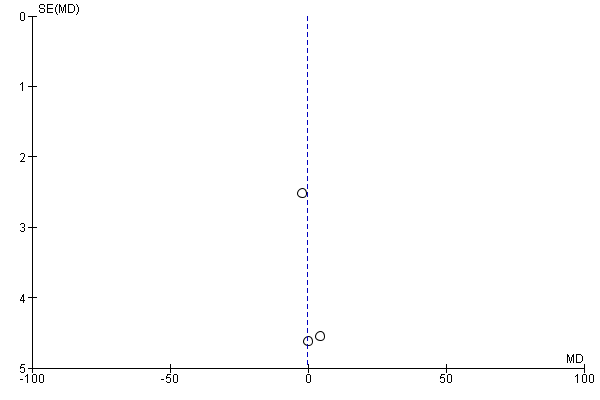

Supplement: Supplementary file 9 — Appendix Fig. 9. Funnel plot of the impact of metformin on LDL; low-density lipoprotein (DOCX 15 KB) [file 431_2023_5169_MOESM9_ESM.docx]

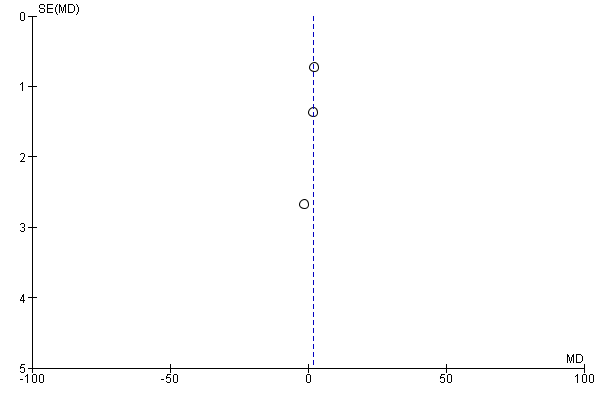

Supplement: Supplementary file 10 — Appendix Fig. 10. Funnel plot of the impact of metformin on HDL; high-density lipoprotein (DOCX 15 KB) [file 431_2023_5169_MOESM10_ESM.docx]

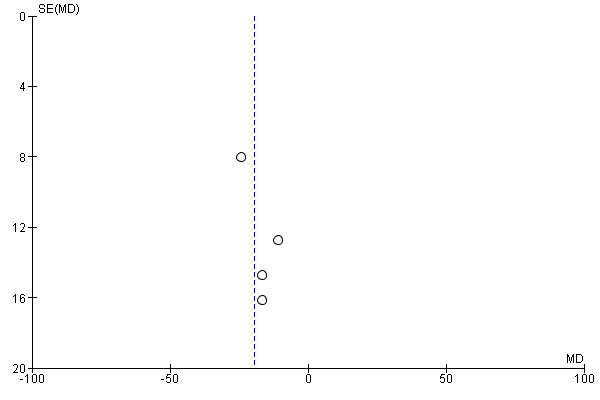

Supplement: Supplementary file 11 — Appendix Fig. 11. Funnel plot of the impact of metformin on TG; triglycerides (DOCX 16 KB) [file 431_2023_5169_MOESM11_ESM.docx]

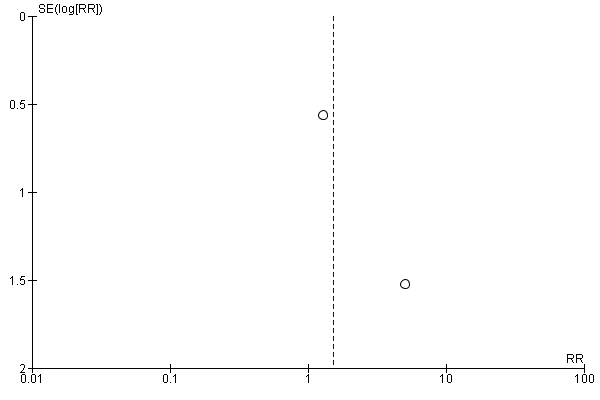

Supplement: Supplementary file 12 — Appendix Fig. 12. Funnel plot of the impact of metformin on the risk for gastrointestinal side effects (DOCX 15 KB) [file 431_2023_5169_MOESM12_ESM.docx]
